# Direct Evidence That Sunbirds’ Gut Microbiota Degrades Floral Nectar’s Toxic Alkaloids

**DOI:** 10.3389/fmicb.2021.639808

**Published:** 2021-03-11

**Authors:** Mohanraj Gunasekaran, Beny Trabelcy, Ido Izhaki, Malka Halpern

**Affiliations:** ^1^Department of Evolutionary and Environmental Biology, Faculty of Natural Sciences, University of Haifa, Haifa, Israel; ^2^Department of Biology and Environment, Faculty of Natural Sciences, University of Haifa, Tivon, Israel

**Keywords:** nicotine, anabasine, dysbiosis, sunbird, gut microbiota, secondary metabolites, *Nicotiana glauca*, tobacco tree

## Abstract

Orange-tufted sunbirds (*Cinnyris osea*) feed on the nectar of the tobacco tree (*Nicotiana glauca*) which contains toxic pyridine alkaloids characterized by high concentrations of anabasine and much lower concentrations of nicotine. We aimed at determining whether the gut microbiota of sunbirds harbors bacterial species that enable the birds to cope with these toxic alkaloids. An *in vivo* experiment that included 12 birds showed that inducing dysbiosis in sunbirds’ guts by the addition of sulfamethoxazole and trimethoprim, significantly reduced the birds’ ability to degrade anabasine (*n* = 3) compared to control birds (*n* = 3) with undisturbed microbiota. Sunbirds whose gut bacterial communities were altered by the antibacterial agents and who were fed with added nicotine, also showed a lower percentage of nicotine degradation (*n* = 3) in their excreta compared to the sunbirds with undisturbed microbiota (*n* = 3), though this difference was not significant. In an *in vitro* experiment, we studied the ability of *Lactococcus lactis*, *Enterobacter hormaechei*, *Chryseobacterium gleum*, *Kocuria palustris*, and *Methylorubrum populi* that were isolated from sunbirds’ excreta, to degrade anabasine and nicotine. By using gas chromatography-mass spectrometry (GC-MS) analysis, we successfully demonstrated, for the first time, the ability of these species to degrade the focal secondary metabolites. Our findings demonstrate the role of gut bacteria in detoxifying toxic secondary metabolites found in the *N. glauca* nectar. The degradation products may supply the birds with nitrogen which is scarce in nectar-rich diets. These findings support another role of bacteria in mediating the interactions between plants and their pollinators.

## Introduction

Microbiota are a collection of microorganisms that inhabit a specific environment, while a microbiome is a collection of genes and genomes of members of a microbiota (Marchesi and Ravel, 2015). Gut microbiota play an important role in the ecology and evolution of their hosts (McFall-Ngai et al., 2013). The study of avian gut microbial diversity and function focuses mostly on commercial bird species such as turkeys and chickens (Grond et al., 2018). Several studies examined the gut microbiota of passerine birds (Lombardo et al., 1996; Mills et al., 1999; Klomp et al., 2008; Benskin et al., 2010), whereas other studies examined their microbiomes (Bagley Lewis, 2015; Kropáčková et al., 2017; Bodawatta et al., 2018; Michel et al., 2018; Lee et al., 2019). In general, the gut of birds is relatively small and under selective pressure to allow flight capacity (Nota, 2003). The nectarivorous and frugivorous birds have only vestigial ceca and the hummingbird, a specialized avian nectarivore, has no ceca. Hummingbirds have extremely fast digestion throughout their digestive tract, which could make colonization by bacteria challenging (Kohl, 2012). Therefore, it has been assumed that the guts of nectarivorous birds do not possess the structures to house an intensive microbiota, presumably needed for effective digestion. Nevertheless, Preest and Beuchat (2003) isolated bacteria from Anna’s hummingbird, and Lee et al. (2019) and Gunasekaran et al. (2020) molecularly analyzed the microbial community of hummingbirds and sunbirds excreta, respectively. These studies suggested that although nectarivorous birds lack large ceca, they possess a diverse bacterial community (278 ASVs) in their gut (Gunasekaran et al., 2020).

Toxic plant secondary metabolites in flower nectar may play a role as mediators in mutualistic plant–animal interactions. It was suggested that secondary metabolites in nectar are actually more beneficial than harmful (Adler, 2000; Stevenson et al., 2017). For example, the “direct toxicity hypothesis” proposes that plant secondary metabolites deter inefficient pollinators and nectar robbers (Detzel and Wink, 1993; Adler, 2000), thus, filtering out nectar robbers and allowing only efficient pollinators to feed on nectar. It was demonstrated that the gut microbiota of the Greater Sage-Grouse which feeds on sagebrush that contains phenols (Wilt et al., 1992; Kohl et al., 2016) and monoterpenes (Wilt and Miller, 1992; Wilt et al., 1992; Kelsey et al., 2020) detoxify these plant secondary metabolites. Degradation of terpenes by the gut microbial communities was also observed in mountain pine beetles (*Dendroctonus ponderosae*) which feed on terpene-rich conifer plants (Adams et al., 2013). Gut microbes of insects that feed on coffee plants mediated detoxification of caffeine (Ceja-Navarro et al., 2015). Nevertheless, nectar consumers must cope not only with the toxic compounds in the nectar but also with the low content of nitrogen in the nectar. It was suggested that the end products of alkaloid degradation may contribute to the nitrogen balance of nectar consumers (Kohl et al., 2016). Bacterial species that degrade nicotine release end products like methylamine which are degraded to ammonium and can be used as a nitrogen source (Ganas et al., 2008; Taubert et al., 2017). Similarly, our studies on frugivores that hydrolyze toxic plant glucosides (Lessner et al., 2015) and on nicotine- and anabasine-degrading bacteria from sunbirds’ gut suggested that gut microbiota of nectarivorous birds may detoxify the toxic compounds in nectar (Gunasekaran et al., 2020).

The orange*-*tufted sunbird (*Cinnyris osea*) is a small passerine bird with a long, slender, decurved bill (1.4–2.0 cm in length), and a long tongue, which allows it to feed mainly on floral nectar and arthropods (Markman et al., 2004). Sunbirds have high sugar-absorption efficiencies despite the rapid speed that food moves through their gut (Roxburgh and Pinshow, 2000). In Israel, the floral nectar of the tobacco tree (*Nicotiana glauca*) is widely used by sunbirds as a food resource (Tadmor-Melamed et al., 2004). As detected for other *Nicotiana* species, its nectar contains toxic pyridine alkaloids (Kessler et al., 2012), but in contrast to the other studied *Nicotiana* species, its predominant pyridine alkaloid is anabasine (5.0 ± 0.8 ppm) rather than nicotine (0.50 ± 0.12 ppm) (Tadmor-Melamed et al., 2004). *N. glauca* is native to Argentina and Bolivia and has been found as an invasive species across the globe, including Australia, other parts of South America, Hawaii, South Africa, the east, and north Mediterranean region including Israel (Ollerton et al., 2012). This plant species depends on pollinating vectors because its flower stamens are shorter than its stigma and has a relatively long corolla; hence, the pollination of *N. glauca* depends mainly on birds with long bills like hummingbirds and sunbirds (Galetto and Bernardello, 1993).

In a previous study (Gunasekaran et al., 2020), we found a significant change in the microbiota composition and diversity in the excreta of sunbirds fed on anabasine and nicotine compared to control birds that were not fed with these secondary metabolites. We also showed that the abundance of nicotine- and anabasine-degrading bacteria in the excreta of birds from the treatment group was significantly higher than in the control. Here we aimed at better understanding the role of sunbirds’ gut microbiota in detoxifying the toxic pyridine alkaloids anabasine and nicotine by directly studying the ability of the sunbirds’ gut microbiota to degrade them. To achieve this goal, we used both *in vivo* and *in vitro* approaches. In the *in vivo* experiment, we studied the ability of dysbiosis vs. undisrupted sunbirds’ gut-associated microbiota to degrade anabasine and nicotine. In an *in vitro* study, we examined the ability of five isolates from our previous study (Gunasekaran et al., 2020), to indeed degrade anabasine and nicotine. Thus, here we provide evidence that the gut microbiota of sunbirds can indeed degrade toxic pyridine alkaloids.

## Materials and Methods

### *In vivo* Dysbiosis Experiment

#### Studied Organisms

We used the orange*-*tufted sunbird as the study organism. Eighteen adult sunbirds were captured using mist nets between December 2018 and January 2019. Each bird was held in a separate cage in a room with controlled temperature (25°C) and 12:12h light:dark conditions. Birds were fed with artificial nectar (Sunbird nectar special formula for Nectariniidae; Aves & Avian, Lot nr IS240718; Reg.nr. NL113333, Raalte, Netherlands). The *in vivo* dysbiosis experiment was performed 2 weeks after the birds were captured. Therefore, we assume that anabasine- and nicotine-degrading bacteria were introduced to these birds when they were free ranging in their natural habitat and foraging on *N. glauca* nectar. Birds were released at the end of the experiment.

#### Ethics Statement

All relevant guidelines and regulations were maintained during the experiments. The sunbirds were captured in Israel with permission from the Israel Nature and Park Authority (permit #2016/41432). All experimental laboratory procedures and animal care were approved by the Committee of Animal Experimentation of the University of Haifa (permit #477/16).

#### Antibacterial Agent Effects

The effectivity of antibacterial agents was examined in a preliminary study on six birds, by treating the birds with different antibiotics and antibacterial agents in various concentrations. The combination of sulfamethoxazole and trimethoprim (Sigma Aldrich, Rehovot, Israel) was found to be the most effective in reducing the bacterial load in the birds’ excreta (Supplementary Figure 1). The birds that were tested in this preliminary experiment were not used in the dysbiosis experiment described below.

#### Dysbiosis Experiment

**FIGURE 1:**
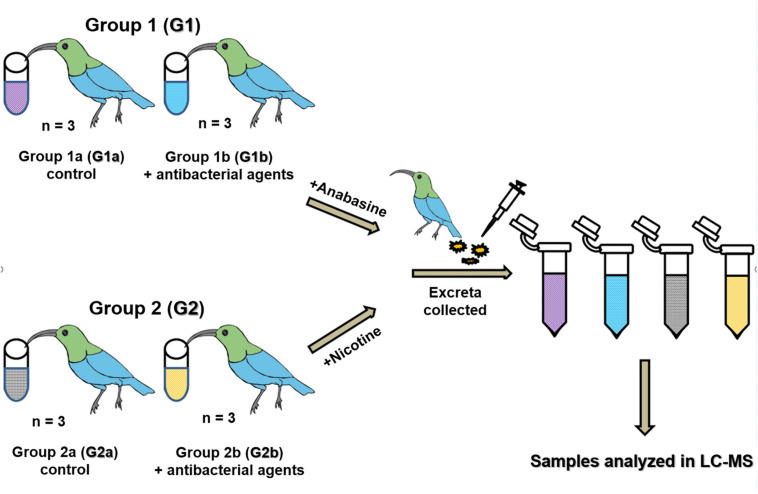
Dysbiosis experiment. Sunbirds were divided into two groups (G1 and G2) which were further divided into two sub-groups a and b. All birds were fed with artificial nectar. A mixture of sulfamethoxazole and trimethoprim was mixed with the artificial nectar to a final concentration of 8 and 1.6 mg/ml, respectively, and fed to the birds in groups G1b and G2b for 72 h. Then, anabasine was added to all the birds in group G1 and nicotine was added to all the birds in group G2. The addition of the antibacterial agents to the food of subgroups G1b and G2b continued in parallel to the addition of the pyridine alkaloids, until the end of the experiment. Excreta samples were collected for anabasine and nicotine degradation analysis in the LC-MS after 24 and 72 h.

Twelve naïve adult sunbirds were adapted to laboratory conditions for 2 weeks and then randomly divided into two groups (G1 and G2). Then, each group was further subdivided into two subgroups (G1a and G1b and G2a and G2b) (Figure 1). Both groups (G1 and G2) were fed with artificial nectar (as described above). An antibacterial agent mixture of sulfamethoxazole and trimethoprim was mixed with the artificial nectar food to final concentrations of 8 and 1.6 μg/ml sulfamethoxazole and trimethoprim, respectively. The birds from groups G1b and G2b were fed with this antibacterial agent mixture for 72 h. After 72 h, the diet of group G1 was changed to artificial nectar, with the addition of anabasine, and the diet of group G2 was changed to artificial nectar, with the addition of nicotine. Subgroups G1b and G2b continued to receive the antibacterial agent treatment as well (Figure 1). The concentrations of anabasine and nicotine (Sigma Aldrich, Rehovot, Israel) were added according to their natural concentrations in *N. glauca* floral nectar (5 and 0.5 ppm, respectively) (Tadmor-Melamed et al., 2004). 24 and 72 h after the addition of anabasine or nicotine, excreta samples were collected (Figure 1) and stored at −20°C for further analysis of the determination of anabasine and/or nicotine concentrations in the samples.

The volume of the consumed food and the volume of the excreta were measured for each bird. Food volume was measured once per day. Excreta volume was measured every hour for 24 h (Supplementary Table 1).

#### Dysbiosis Sample Analyses

Excreta samples were collected in 1.5 ml Eppendorf vials and stored at −20°C. For the analyses, samples were thawed on ice and (0.1 ml) were diluted (1:5) with five volumes of 80% methanol solution and vortexed at 4°C for 2 min before centrifugation (10,000 *g* for 5 min) to remove precipitations. An aliquot of the supernatant was transferred to a sterile screw-capped vial for liquid chromatography-mass spectrometry (LC-MS) analysis (Agilent Technologies, Waldbronn, Germany). The efficiency of nicotine and anabasine degradation was calculated for each bird separately, based on the input and output volumes of nicotine and anabasine, respectively (Supplementary Table 1).

#### Nicotine and Anabasine Quantitation in Excreta Samples

The determination of nicotine and anabasine concentrations in the excreta samples of the sunbirds was performed using LC coupled to MS–MS in positive ionization mode. The LC-MS is a very sensitive analytical technique for the quantification of easily ionizable organic compounds. All 24 excreta samples were analyzed using LC-MS. The representative ions (M+H) of the target compounds (Supplementary Table 2) were detected using the “find compound by formula” function and analyzed by Masshunter qualitative and quantitative analysis software version B.07.00 (Agilent Technologies). Compounds were identified by comparison of the exact mass and retention time to purchased standards (Sigma–Aldrich, Darmstadt, Germany, N3876, ≥99% pure, GC quality). For quantitation, purchased analytical standards were dissolved in a stock solution at 1,000 ppm and then diluted with 80% methanol to a series of concentrations: 0.0039, 0.0079, 0.0156, 0.03125, 0.0625, 0.125, 0.25, 0.5, and 1 ppm in triplicates for generating a standard curve (R^2^ > 0.98 for anabasine and nicotine). Standards and samples were also subjected to tandem MS for optimal identification with 163.123 as a precursor ion. Nicotine and anabasine quantitation in the artificial nectar samples (input: 0.5 ppm nicotine, 5.0 ppm anabasine) and the excreta (output) were determined and quantified by comparing to standards. The concentration of the pyridine alkaloids input in the food, and the output in the excreta were calculated, and their concentrations were determined in LC-MS (Supplementary Table 2). The percentages of nicotine and anabasine degradation were determined using the following equations:

Input alkaloid concentration

$$=\frac{\overset{\mathrm{Food}\,\mathrm{input}\,\left(\mathrm{ml}\right)\times \mathrm{Initial}\,\mathrm{concentration}\,\mathrm{of}}{\mathrm{the}\,\mathrm{alkaloid}\,\mathrm{in}\,\mathrm{the}\,\mathrm{food}}}{\mathrm{Total}\,\mathrm{volume}\,\mathrm{input}\,\left(\mathrm{food}+\mathrm{water}\right)}$$

Percentage of alkaloid left in the excreta

$$=\frac{\mathrm{Concentration}\,\mathrm{of}\,\mathrm{alkaloid}\,\mathrm{in}\,\mathrm{excreta}}{\mathrm{Input}\,\mathrm{alkaloid}\,\mathrm{concentration}}\times 100$$

Percentage of alkaloid degradation

=100-Percentage⁢of⁢alkaloid⁢left⁢in⁢the⁢excreta

### *In vitro* Analysis of Nicotine and Anabasine Degradation

#### Bacterial Strains

Our study focused on the analyses of sunbirds excreta as this sample type provides the most accurate assessment of the colon microbiome without the need to sacrifice the birds (Videvall et al., 2018). Five bacterial strains that were isolated and identified from sunbirds’ excreta samples in a previous study (Gunasekaran et al., 2020) were analyzed in the current study for their ability to degrade anabasine and nicotine. The strains’ isolation and identification were performed by Gunasekaran et al. (2020) on M9 minimal medium (m6030, Sigma–Aldrich, St. Louis, MO, United States) with the addition of anabasine or nicotine as sole carbon and nitrogen sources. The isolation procedure is described in detail by Gunasekaran et al. (2020). Gunasekaran et al. (2020) isolated a total of 146 anabasine/nicotine-degrading bacterial strains. These strains belonged to 14 different genera. Seven of these genera were never described before in the literature as anabasine and/or nicotine degraders, and thus, we have chosen five representative species from these genera to study toward understanding their ability to degrade these two pyridine alkaloids. The relative abundances in the sunbirds’ excreta, of the bacterial genera that were capable of degrading anabasine and nicotine are summarized in Supplementary Table 3 (data from Gunasekaran et al., 2020). The most prominent genus was *Lactococcus*, which was always more abundant compared to the other microbiota that inhabited the birds’ excreta. Its abundances in sunbirds’ excreta, in the previous study after 4 and 7 weeks of feeding with anabasine and nicotine were 49.5 and 28.1%, compared to 35.8 and 14.3% in the control birds, respectively (Gunasekaran et al., 2020). All other genera comprised a much lower percentages of the total bacterial community (∼<1%; Supplementary Table 3), and thus, the following bacterial species; *Lactococcus lactis* (MK348788.1), *Enterobacter hormaechei* (MK348783.1), *Chryseobacterium gleum* (MK348808.1), *Kocuria palustris* (MK348831.1), and *Methylorubrum populi* (MK348790.1) were chosen for the *in vitro* analysis of anabasine and nicotine degradation.

Bacterial strains were grown on Luria broth (LB, HiMedia, Mumbai). Exponential phase culture was harvested by centrifugation and washed twice with M9 medium. Sedimented cultures were then resuspended in freshly prepared M9 medium with the addition of 0.1% anabasine or 0.1% nicotine (Sigma–Aldrich, Darmstadt, Germany) and incubated overnight at 28°C with aeration.

The overnight M9 culture medium with the addition of 0.1% anabasine was then harvested, and its optical density was adjusted to 0.1 OD_600_. One milliliter of 0.1 OD_600_ bacterial culture was inoculated in freshly prepared 100 ml M9 medium with 0.1% anabasine. One milliliter culture was harvested every hour, serially diluted in saline water, and plated on LB agar. LB plates were incubated at 28°C for 24 h, and colony-forming units (CFUs) were counted and calculated per ml. Simultaneously, 1 ml was also taken from the culture flask and centrifuged, and then the supernatant was extracted with an equal volume of dichloromethane (Sigma–Aldrich, Darmstadt, Germany). The extracted solvent was used for further gas chromatography-mass spectrometry (GC-MS) analysis. To track nicotine degradation, samples were treated in the same way using the same procedure, except that the M9 medium was prepared with the addition of 0.1% nicotine. Each experiment was carried out in three independent replicates.

#### GC-MS Analysis

The extracted solvent was concentrated by vacuum, and a sample of 5 μl was used for GC-MS analysis (Agilent GC 7890A connected to MS 5975C). The operation parameters were as follows: the chemical separation of the sample was carried out on a column (Restek Rxi-5sil MS, 30 m, ID 0.25 mm, 0.25 μm coating, Restek Corporation, Bellefonte, PA, United States); the oven temperature starting point was 80°C for 5 min and then it was increased at the rate of 4°C/min until it reached 144°C. Next, the rate of the increased temperature was 50°C/min up to 300°C for 1 min. To generate a standard curve for nicotine or anabasine, the purchased standards were dissolved in distilled water to a series of concentrations between 0.01 and 0.2% (in triplicates).

### Statistical Analyses

A one-tailed *t*-test for two independent samples was applied to compare the proportions of anabasine and nicotine degradation between the different treatments. Percentages of degraded anabasine/nicotine were arcsine transformed, and all groups were normally distributed according to the Shapiro–Wilk test (*p* > 0.05). The equality of variances was not violated according to Levene’s test (*p* > 0.05). A paired samples *t*-test was used to compare the proportions of anabasine and nicotine degradation at 24 vs. 72 h after introducing the birds to anabasine or nicotine. All statistical analyses were performed using IBM SPSS 25. All results are presented as mean ± standard error of the mean (SEM).

## Results

### Dysbiosis *in vivo* Experiment

#### Antibacterial Treatment

The combination of sulfamethoxazole and trimethoprim at final concentrations of 8 and 1.6 μg/ml, respectively, reduced the bacterial counts by almost three orders of magnitudes (from 3.8 × 10^7^ to 8.0 × 10^4^) after 72 h of treatment, compared to the control (Supplementary Figure 1).

Twelve birds were used for the *in vivo* experiment (Figure 1). These birds were divided into two groups; the birds in group G1 were tested for their ability to degrade anabasine, while the birds in group G2 were tested for their ability to degrade nicotine. At the beginning of the experiment, subgroups G1b and G2b were treated with a combination of sulfamethoxazole and trimethoprim. After 72 h of the antibacterial agent treatment, anabasine was added to the diets of all birds in group G1 and nicotine was added to the diets of all birds in group G2 (Figure 1). The addition of the antibacterial agents to the food of subgroups G1b and G2b continued in parallel to the addition of alkaloids, until the end of the experiment (Figure 1). No differences in behavior or physiology were observed in any of the 12 birds that participated in the experiment.

**FIGURE 2:**
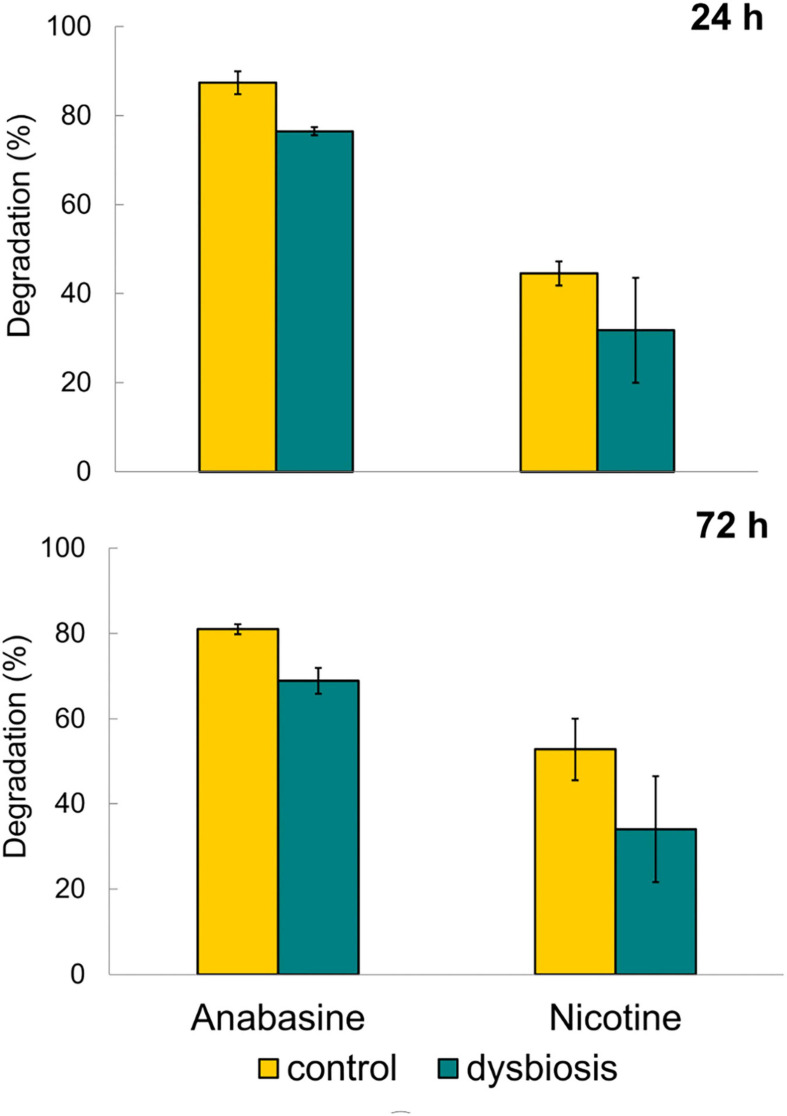
Degradation of anabasine and nicotine after 24 and 72 h of treatment with alkaloids and alkaloids + antibacterial agents. *n* = 3 birds in each of the four treatments (see also Figure 1). Results are presented as mean ± SEM.

Control sunbirds with undisturbed microbiota composition (G1a) exhibited significantly higher degradation of anabasine compared to the sunbirds that were in a dysbiotic state (G1b), both after 24 h (87.4% ± 4.4 and 76.5% ± 1.5, respectively, *t*_4_ = 3.61, *p* = 0.011, Figure 2) and after 72 h (81.0% ± 2.1 and 68.9% ± 5.3, respectively, *t*_4_ = 3.88, *p* = 0.009, Figure 2). Thus, anabasine degradation in the dysbiotic sunbirds was significantly less effective compared to the sunbirds with normal microbiota composition (Figure 2 and Supplementary Table 4).

Nicotine degradation in the excreta of the birds with undisturbed microbiota (G2a) (control group) was higher than in the excreta of the dysbiotic sunbirds (G2b), both after 24 h (44.5% ± 4.7, *n* = 3; 31.8% ± 20.4, *n* = 3, respectively) as well as after 72 h (group G2a, 52.8% ± 12.5 and group G2b 34.1% ± 21.5, respectively, Figure 2). However, the results were not statistically significant (24 h: *t*_4_ = 1.11, *p* = 0.17; 72 h: *t*_4_ = 1.34, *p* = 0.13). This may be due to the large variation in nicotine concentrations in the excreta of the dysbiotic sunbirds (one of the birds was an outliner; see Bird 4 in group G2b, Supplementary Table 5).

No significant differences in anabasine degradation were found between samples from 24 and 72 h for both bird groups (with undisturbed microbiota: *t* = 3.44, *df* = 2, *p* = 0.075; with dysbiosis: *t* = 3.47, *df* = 2, *p* = 0.074). Similar results were observed for nicotine degradation at 24 and 72 h for both the control birds with undisturbed microbiota (*t* = 1.68, *df* = 2, *p* = 0.24) and for the birds with dysbiosis (*t* = 2.17, *df* = 2, *p* = 0.16).

### *In vitro* Alkaloid Degradation

Recently, Gunasekaran et al. (2020) isolated different bacterial species that were able to grow with anabasine and nicotine as sole carbon and nitrogen sources. The following species: *M. populi*, *L. lactis*, *E. hormaechei*, *C. gleum*, and *K. palustris*, not previously described as anabasine or nicotine degraders, were chosen for the *in vitro* study of anabasine and nicotine degradation.

#### Anabasine Degradation

**FIGURE 3:**
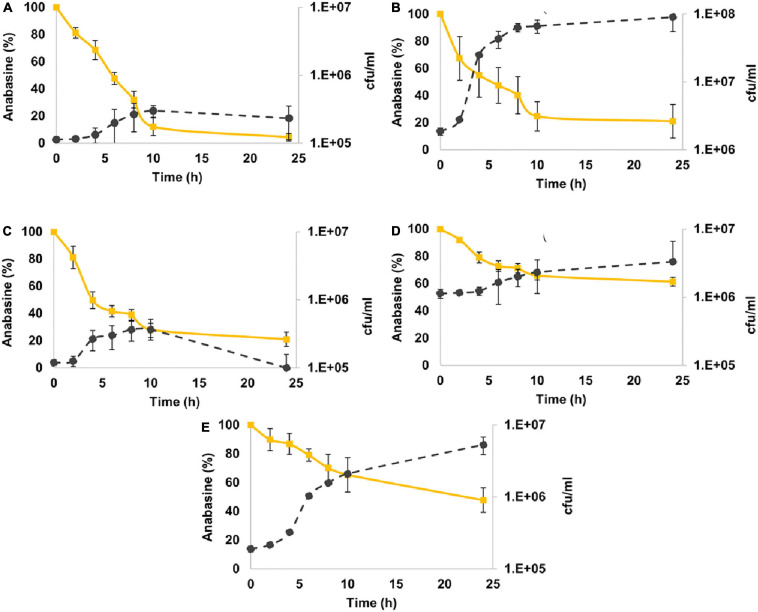
Anabasine degradation by sunbirds’ gut bacterial isolates. **(A)** *Methylorubrum populi* (MK348790.1), **(B)** *Chryseobacter gleum* (MK348808.1), **(C)** *Enterobacter hormaechei* (MK348783.1), **(D)** *Lactococcus lactis* (MK348788.1), and **(E)** *Kocuria palustris* (MK348831.1). Anabasine percentage curves are in yellow, while the number of bacterial colonies per ml (cfu/ml) curves is in black. Results are presented as mean ± SEM.

All bacterial strains reduced anabasine concentration (Figure 3). *M*. *populi* demonstrated the best performance of anabasine degradation compared to the other strains by degrading 95% of the anabasine. It was followed by *C. gleum* and *E. hormaechei*, with 79% anabasine degradation. *K. palustris* showed a moderate utilization of anabasine with 52% degradation, while *L. lactis* degraded only 38% of the anabasine (Figure 3).

#### Nicotine Degradation

**FIGURE 4:**
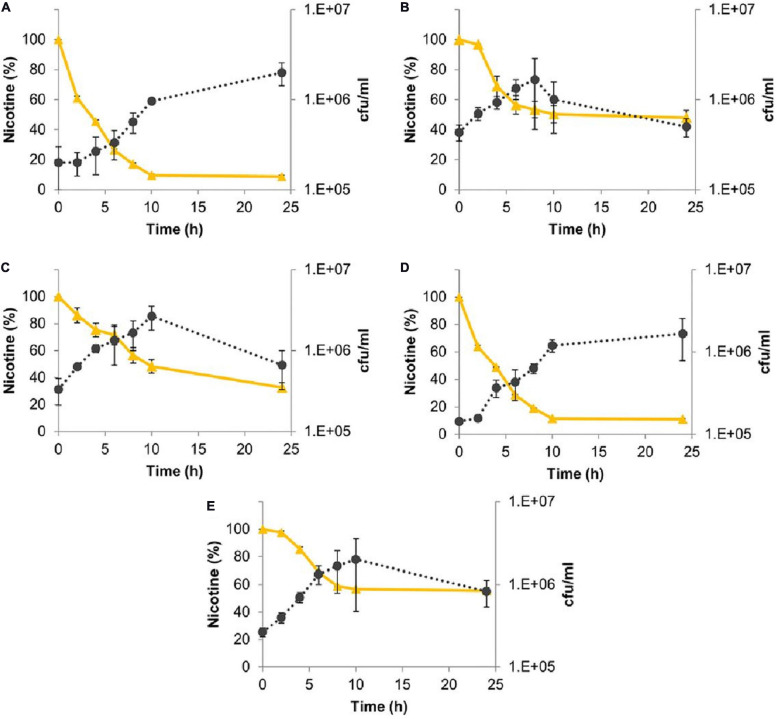
Nicotine degradation by sunbirds’ gut bacterial isolates. **(A)** *Methylorubrum populi* (MK348790.1), **(B)** *Chryseobacter gleum* (MK348808.1), **(C)** *Enterobacter hormaechei* (MK348783.1), **(D)** *Lactococcus lactis* (MK348788.1), and **(E)** *Kocuria palustris* (MK348831.1). Nicotine percentage curves are in yellow, while the number of bacterial colonies per ml (cfu/ml) curves is in black. Results are presented as mean ± SEM.

All the five bacterial species were also able to efficiently degrade nicotine (Figure 4). *M*. *populi* and *L. lactis* exhibited 91 and 89% degradation, respectively. *E. hormaechei*, *C. gleum*, and *K. palustris* were less effective with 68, 53, and 45% of nicotine degradation, respectively (Figure 4).

**FIGURE 5:**
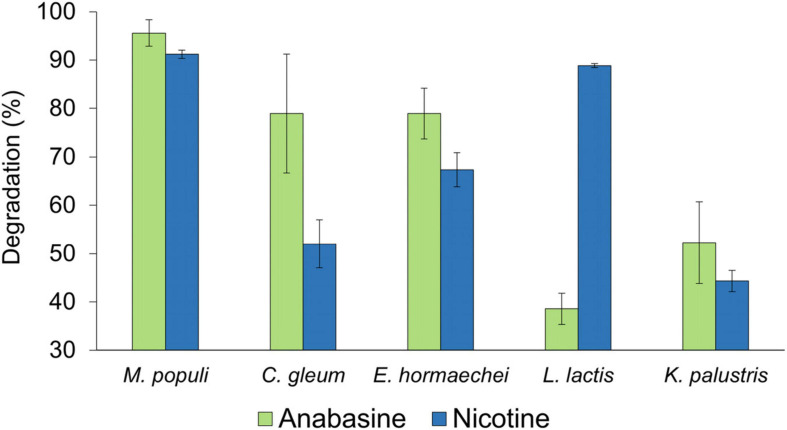
Anabasine and nicotine degradation activities by the five sunbird bacterial isolates, *Methylorubrum populi*, *Chryseobacter gleum*, *Enterobacter hormaechei*, *Lactococcus lactis*, and *Kocuria palustris*. Results are presented as mean ± SEM.

The different bacterial species showed different efficiencies in degrading the two pyridine alkaloid compounds (Figure 5). For both alkaloids, their concentrations were reduced as long as the bacterial strains were in their logarithmic growth phase, and the maximal degradation activity was observed after about 10 h incubation. No significant differences were found in the ability of *M. populi* to degrade anabasine (95%) and nicotine (90%) (*t* = 1.52, *df* = 4, *p* = 0.20, Figure 4), nor were significant differences found in the abilities of *C. gleum*, *E. hormaechei*, and *K. palustris* to degrade anabasine vs. nicotine (*t* = 2.03, *df* = 4, *p* = 0.11; *t* = 1.84, *df* = 4, *p* = 0.14; *t* = 0.91, *df* = 4, *p* = 0.41, respectively). Significant differences were found between anabasine and nicotine degradation by *L. lactis* (*t* = 15.40, *df* = 4, *p* = 0.0001, Figure 5) which very efficiently degraded nicotine (89%) but much less effectively degraded anabasine (38%).

## Discussion

The presence of toxic pyridine alkaloids such as anabasine and nicotine in the floral nectar of the tobacco tree (*N. glauca*) raises various questions on their potential evolutionary role within the interactions between plants and their nectar consumers (Singaravelan et al., 2005; Lerch-Henning and Nicolson, 2013; Kaczorowski and Markman, 2016; Stevenson et al., 2017; Gunasekaran et al., 2020). One hypothesis is that toxic compounds deter nectar thieves and selectively allow only efficient pollinators to feed on nectar, thus promoting plant fitness (Adler, 2000; Lerch-Henning and Nicolson, 2013; Stevenson et al., 2017).

In a previous study (Gunasekaran et al., 2020), we showed that the microbiota composition and diversity in the excreta of sunbirds fed with anabasine and nicotine were significantly different from that of control birds. We have also shown that the abundance of nicotine- and anabasine-degrading bacteria in the excreta of birds from the anabasine and nicotine treatment group was significantly higher than in the control (Gunasekaran et al., 2020). To further test our hypothesis that the sunbirds’ gut microbiota facilitates the degradation of toxic pyridine alkaloids consumed from tobacco tree nectar, we induced dysbiosis in sunbirds’ gut microbiota. The dysbiotic sunbirds were significantly less efficient than control birds at degrading anabasine, which is the predominant toxic compound in *N. glauca* nectar (Figure 2). The same phenomenon was observed for nicotine degradation, but results were not statistically significant, likely due to small sample size and one bird that was as an outliner (Supplementary Table S5).

A previous study on the presence of secondary metabolites in the excreta of two nectarivorous birds, the white-bellied sunbird (*Cinnyris talatala*) and the Cape white-eye (*Zosterops virens*), in South Africa, suggested different detoxification processes in each of these two species (Lerch-Henning and Nicolson, 2013). Interestingly, this may be a result of different gut bacterial compositions. In Israel, the orange*-*tufted sunbirds are one of the pollinators of the tobacco tree. Our previous study demonstrated that the presence of anabasine and nicotine in the nectar’ affects the bacterial community structure in the sunbirds’ gut (Gunasekaran et al., 2020). Furthermore, we have isolated several anabasine- and nicotine-degrading bacterial species from the excreta of sunbirds that were fed on *N. glauca* nectar (Gunasekaran et al., 2020). These species belonged to the genera *Pseudomonas*, *Delftia*, *Acinetobacter*, *Enterobacter*, *Stenotrophomonas*, *Klebsiella*, *Chryseobacterium*, *Exiguobacterium*, *Lactococcus*, *Brevibacterium*, *Kocuria*, *Methylorubrum*, *Roseomonas*, and *Sphingobacterium*. Of them, *Pseudomonas*, *Delftia*, *Stenotrophomonas*, *Klebsiella*, *Brevibacterium*, and *Sphingobacterium* were already reported as nicotine degraders (Ruan et al., 2006; Ma, 2012; Sabourmoghaddam et al., 2015; Gaekwad and Vinchurkar, 2018; Hu et al., 2019).

In general, nectarivorous birds may face the problem of nitrogen balance as the floral nectar, particularly of hummingbird pollinated plants, contain low levels of proteins (Gartrell, 2000; López-Calleja et al., 2003; Nicolson and Fleming, 2003; Brice and Grau, 2010; Michel et al., 2013). Also, during our pilot study, we found that the nectar of the tobacco tree contains only 12.0 ± 5.0 μg ml^–1^ of proteins, which is very low in comparison with the mean value of total protein in floral nectars (ca. 100 μg ml^–1^) (Nicolson and Fleming, 2003). Nicotine degradation yields by-products like maleamic acid and γ-aminobutyrate. The nicotine catabolism by *Ochrobacterium* species yields maleamic acid as a byproduct, which is further catabolized to maleic acid and ammonia (Yu et al., 2015). Similarly, *Arthrobacter nicotinovorans* catabolizes nicotine to γ-aminobutyrate, which is further degraded to succinic semialdehyde releasing free ammonia (Gurusamy and Natarajan, 2013). Similarly, anabasine and nicotine degradation by bacteria from sunbirds’ guts releases free ammonia which may be used by the birds as a nitrogen source. Several avian species were reported to possess intestinal bacteria that may contribute to their nitrogen balance, for example, the presence of uric-acid-degrading bacteria in the ceca of duck and guinea fowl (Barnes, 1972). This was also reported for the insect pest *Hypothenemus hampei* whose microbiota play a role in caffeine degradation (Ceja-Navarro et al., 2015).

Our results show that the species isolated from the sunbirds’ excreta do indeed degrade both anabasine and nicotine with high efficiency (Figures 3, 4). Nicotine degradation has previously been reported; however, reports on bacterial degradation of anabasine are very rare. Nicotine degradation was reported for the following bacterial species and genera; *Pseudomonas putida* (isolated from marine sediments) (Li et al., 2017), *Ochrobactrum* sp. (Yu et al., 2015), *Massilia*, *Erwinia*, *Brevundimonas*, and *Paenibacillus* (isolated from tobacco rhizosphere and phyllosphere) (Lei et al., 2015), and *Arthrobacter*, *Sphingobacterium*, *Pseudoxanthomonas*, and *Delftia* (identified in soils of a tobacco plantation) (Ma, 2012). Nevertheless, our study has demonstrated that nicotine-degrading bacteria inhabit the guts of sunbirds. As far as we know, this is the first report of these five genera’s ability to degrade anabasine and nicotine (Figures 3, 4).

There are very few reports of bacterial degradation of anabasine. The genus *Arthrobacter* and *Pseudomonas* were found to degrade anabasine from tobacco waste (Eberhardt, 1995). All of our five studied isolates exhibited anabasine degradation abilities (Figure 3). The best degradation ability was observed in *M. populi* with 95% of degradation. *C. gleum* and *E. hormaechei* both showed 79% of anabasine degradation.

Among these five isolates, *M. populi* (91%, degradation) also exhibited the highest capacity to degrade nicotine (91%), followed by *L. lactis* (89%) (Figures 4A,D, respectively). Symbiotic relationships have been reported between *Methylorubrum* species and plants (Van Aken et al., 2004). Members of this genus are known to metabolize various toxic organic chemicals, such as methyl bromide (Goodwin et al., 2001), dichloromethane (Doronina et al., 2000), methylated amines (Trotsenko et al., 2001), ethylated sulfur-containing compounds (De Zwart et al., 1996), and thiocyanate (Wood et al., 1998). *L. lactis* is a beneficial organism in the food industry and is used as a microbial cell factory for the production of industrially important products with great economic values (Song et al., 2017). *L. lactis* was the most abundant Amplicon Sequence Variant (ASV) in the gut of sunbirds (Gunasekaran et al., 2020), and the second most efficient nicotine degrader in the current study (Figure 4D).

Our study supports the role of bacteria in shaping the interactions between plants and their pollinators. Here we demonstrate that sunbirds’ gut bacterial isolates effectively degrade anabasine and nicotine. We found that dysbiotic sunbirds were significantly less effective in degrading the toxic alkaloids compared to the control birds. As far as we know, the reported isolates, as well as the dysbiosis experiment to test toxic alkaloid degradation, are the first of their kind. Altogether these results support our hypothesis that the gut bacterial community of the sunbirds degrades the toxic secondary metabolites found in *N. glauca* nectar. These may have a role in nitrogen supply to the sunbirds that feed on very low protein- and nitrogen-content nectar.

An interesting question for future study is whether the ability to detoxify the toxic diet is exclusive to nectarivorous birds. A comparative study with manipulated diets on other bird species, e.g., frugivores, may illustrate if sunbirds host a microbial assemblage that assists in detoxification.

## Data Availability Statement

The raw data supporting the conclusions of this article will be made available by the authors, without undue reservation.

## Ethics Statement

The animal study was reviewed and approved by the Committee of Animal Experimentation of the University of Haifa (permit #477/16, expiration date September 2020).

## Author Contributions

MG, II, and MH conceived and designed the experiments. MG performed laboratory experiments, data collection, analysis, and interpretation of data, and wrote the draft manuscript. BT interpreted and analyzed the GC-MS data. II and MH contributed reagents and materials, helped with data analysis, supervision, and reviewing, and commented on the article. All authors contributed to the article and approved the submitted version.

## Conflict of Interest

The authors declare that the research was conducted in the absence of any commercial or financial relationships that could be construed as a potential conflict of interest.

## Supplementary Material

Supplementary Figure 1 Antibacterial effect of sulfamethoxazole and trimethoprim on the gut microbiota of sunbirds, treated for various time intervals. Three days after starting to treat the birds with a combination of sulfamethoxazole and trimethoprim at final concentrations of 8 and 1.6 μg/ml, respectively, their bacterial load in the excreta was reduced to 0.2% compared to the control (untreated birds).

Supplementary Table 1 Food input and excreta output volumes recorded 24 h after starting to feed the birds with anabasine or nicotine.

Supplementary Table 2 The retention time and the mass of target compounds anabasine and nicotine as observed in the LC-MS analysis.

Supplementary Table 3 Relative abundances of bacterial genera capable of degrading anabasine and nicotine in sunbirds’ excreta that were not previously reported before to degrade these alkaloids. Representatives of these genera were studied in the current study in the *in vitro* experiments. Data were obtained from Gunasekaran et al. (2020). Gunasekaran et al. (2020) studied the microbiota composition of 16 sunbirds that were divided to two groups. The control group (C) was fed with artificial nectar without any additional nutrients, while the treatment group (T) was fed with the same artificial nectar with the addition of anabasine and nicotine (5 and 0.5 ppm, respectively).

Supplementary Table 4 Raw data for calculating anabasine degradation efficiency from sunbirds’ excreta, 24 and 72 h after anabasine addition to the food of birds with undisturbed microbiota (control, G1a) and birds with dysbiotic microbiota (G1b). Anabasine concentrations were measured by LC-MS analysis. *n* = 3 birds for each treatment (see also Figures 1, 2).

Supplementary Table 5 Raw data for calculating nicotine degradation efficiency from sunbirds’ excreta, 24 and 72 h after nicotine addition to the food of birds with undisturbed microbiota (control, G2a) and birds with dysbiotic microbiota (G2b). Nicotine concentrations were measured by LC-MS analysis. *n* = 3 birds for each group treatment (see also Figures 1, 2).
