# Supplementary material for: Direct Evidence That Sunbirds’ Gut Microbiota Degrades Floral Nectar’s Toxic Alkaloids
Source: Front Microbiol. 2021 Mar 11;12:639808. doi: 10.3389/fmicb.2021.639808 (PMC8018289; doi:10.3389/fmicb.2021.639808)
Supplement: Supplementary Figure 1 — Antibacterial effect of sulfamethoxazole and trimethoprim on the gut microbiota of sunbirds, treated for various time intervals. Three days after starting to treat the birds with a combination of sulfamethoxazole and trimethoprim at final concentrations of 8 and 1.6 μg/ml, respectively, their bacterial load in the excreta was reduced to 0.2% compared to the control (untreated birds). [file Data_Sheet_1.pdf]

Supplementary Material

Supplementary Figure

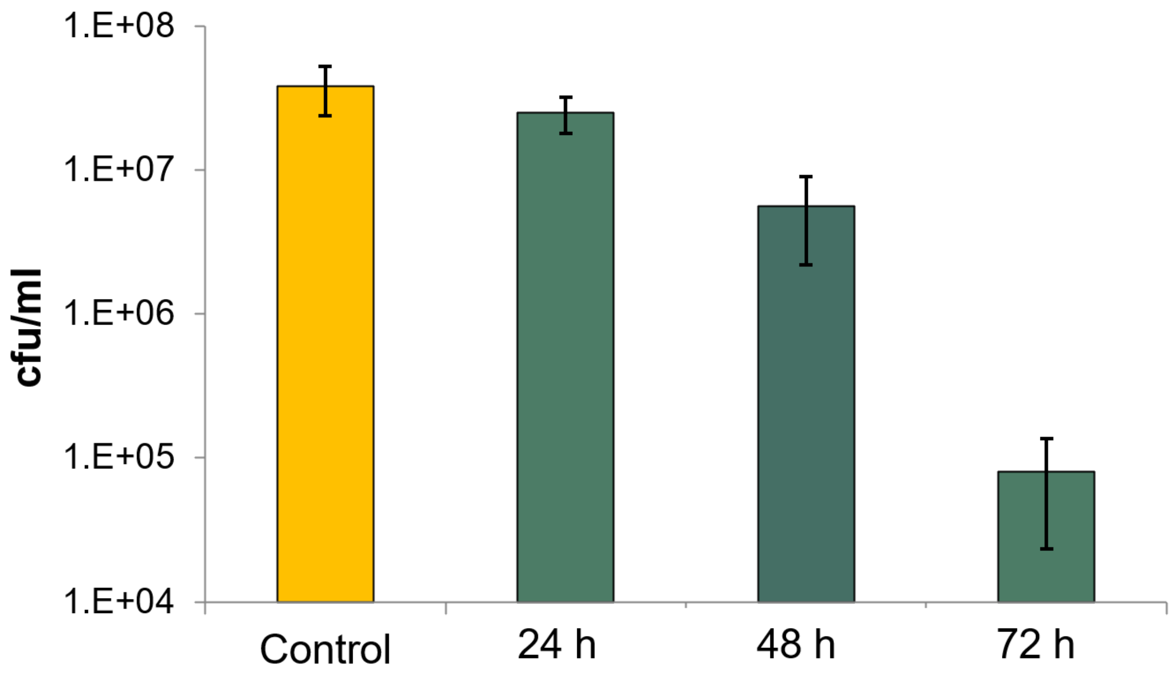

Supplementary Figure 1. Antibacterial effect of sulfamethoxazole and trimethoprim on the gut microbiota of sunbirds treated for various time intervals. Three days after starting to treat the birds with a combination of sulfamethoxazole and trimethoprim at final concentrations of 8µg/ml and 1.6µg/ml, respectively, their bacterial load in the excreta was reduced to 0.2% compared to the control (untreated birds). Results are presented as mean ± SEM.

Supplementary Tables

Supplementary Table 1. Food input and excreta output volumes recorded 24 h after starting to feed the birds with anabasine or nicotine.

Treatment	Bird	Intake (ml)		Output (ml)
		Food	Water	Excreta
**G1a** control + anabasine	Bird 7	40	2.5	4.8
	Bird 8	20	3	4
	Bird 9	25	1	3
**G1b** + anabasine +antibacterial agents	Bird 10	24	1	3
	Bird 11	17	2	2
	Bird 12	21	1	3.5
**G2a** control + nicotine	Bird 1	15	1.5	4
	Bird 2	24	1	5
	Bird 3	20	3	3.5
**G2b** + nicotine +antibacterial agents	Bird 4	14	2	4.5
	Bird 5	17	2	3.9
	Bird 6	20	4	2

Supplementary Table 2. The retention time and the mass of target compounds anabasine and nicotine as observed in the LC-MS analysis.

Compound name	RT min	Formula	Neutral mass	M/Z +
**Anabasine**	3.4	$C_{10}H_{14}N_2$	162.1156	163.123
**Nicotine**	1.9	$C_{10}H_{14}N_2$	162.1156	163.123

RT min, Retention Time per minute
M/Z+, mass-to-charge ratio (for positive ions)

Supplementary Table 3. **Relative abundances of bacterial genera capable of degrading anabasine and nicotine in sunbirds' excreta that were not previously reported before to degrade these alkaloids.** Representatives of these genera were studied in the current study in the *in vitro* experiments. Data was obtained from Gunasekaran et al. (2020)*. Gunasekaran et al. (2020) studied the microbiota composition of 16 sunbirds that were divided to two groups. The control group (C) was fed artificial nectar without any additional nutrients while the treatment group (T) was fed the same artificial nectar with the addition of anabasine and nicotine (5 and 0.5 ppm, respectively). Results are presented as mean ± SEM. W, week; C, control; T, treatment.

Genus	0WC	0WT	2WC	2WT	4WC	4WT	7WC	7WT
Acinetobacter	0.231704 ± 0.0527	0.184708 ± 0.0671	0.023299 ± 0.0038	0.029128 ± 0.0060	0.014617 ± 0.0042	0.011058 ± 0.0048	0.000935 ± 0.0004	0.000546 ± 0.0003
Chryseobacterium	0.000506 ± 0.0005	0	0.000078 ± 7.8E-05	0.000086 ± 8.7E-05	0.000109 ± 7.2E-05	0.000903 ± 0.0007	0	0.000653 ± 0.0004
Exiguobacterium	0.134430 ± 0.0468	0.152816 ± 0.0741	0.080503 ± 0.0308	0.049391 ± 0.0215	0.006562 ± 0.0023	0.001969 ± 0.0010	0.014405 ± 0.0108	0.000676 ± 0.0004
Kocuria	0	0	0	0	0.000533 ± 0.0004	0.000047 ± 4.7E-05	0.000255 ± 0.0002	0.000038 ± 2.6E-05
Lactococcus	0.162420 ± 0.0562	0.073474 ± 0.0421	0.349184 ± 0.0604	0.352911 ± 0.0350	0.143419 ± 0.0355	0.281323 ± 0.0442	0.358921 ± 0.0399	0.495347 ± 0.0449
Methylorubrum	0.000042 ± 4.2E-05	0.000099 ± 8.7E-05	0	0.000005 ± 4.8E-06	0.000446 ± 0.0002	0.000178 ± 0.0001	0	0.000013 ± 1.3E-05
Roseomonas	0	0	0	0	0	0.000074 ± 5.6E-05	0	0

*Gunasekaran, M., Lalzar, M., Sharaby, Y., Izhaki, I., Halpern, M., 2020. The effect of toxic pyridine-alkaloid secondary metabolites on the sunbird gut microbiome. npj Biofilms Microbiomes 6, 1–9. https://doi.org/10.1038/s41522-020-00161-9.

Supplementary Table S4. **Raw data for calculating anabasine degradation efficiency from sunbirds' excreta**, 24 and 72 h after anabasine addition to the food of birds with undisturbed microbiota (control, G1a) and birds with dysbiotic microbiota (G1b). Anabasine concentrations were measured by LC-MS analysis. n = 3 birds for each treatment (see also Fig. 1 and Fig. 2). SEM, standard error of the mean; Conc., concentration.

**Anabasine, 24 h incubation**											
**Treatment**	**Bird ID**	**Food intake (ml)**	**Water intake (ml)**	**Total intake (ml)**	**Initial conc. of anabasine in food (ng/ml)**	**Total anabasine intake conc. (ng/ml)**	**Conc. of anabasine in excreta (ng/ml)**	**Anabasine left in excreta (%)**	**Anabasine degraded (%)**	**Average anabasine degraded (%)**	**SEM**
**G1a** Control	Bird 7	40.00	2.50	42.50	3394.00	3194.35	545.50	17.08	82.92	**87.35**	**2.56**
	Bird 8	20.00	3.00	23.00	3394.00	2951.30	242.50	8.22	91.78		
	Bird 9	25.00	1.00	26.00	3394.00	3263.46	413.00	12.66	87.34		
**G1b** +Antibacterial agents	Bird 10	24.00	1.00	25.00	3394.00	3258.24	736.00	22.59	77.41	**76.46**	**0.89**
	Bird 11	17.00	2.00	19.00	3394.00	3036.74	769.00	25.32	74.68		
	Bird 12	21.00	1.00	22.00	3394.00	3239.73	735.50	22.70	77.30		
**Anabasine, 72 h incubation**											
**Treatment**	**Bird ID**	**Food intake (ml)**	**Water intake (ml)**	**Total intake (ml)**	**Initial conc. of anabasine in food (ng/ml)**	**Total anabasine intake conc. (ng/ml)**	**Conc. of anabasine in excreta (ng/ml)**	**Anabasine left in excreta (%)**	**Anabasine degraded (%)**	**Average anabasine degraded (%)**	**SEM**
**G1a** Control	Bird 7	40.00	2.50	42.50	3394.00	3194.35	681.00	21.32	78.68	**81.04**	**1.19**
	Bird 8	20.00	3.00	23.00	3394.00	2951.30	516.50	17.50	82.50		
	Bird 9	25.00	1.00	26.00	3394.00	3263.46	589.50	18.06	81.94		
**G1b** +Antibacterial agents	Bird 10	24.00	1.00	25.00	3394.00	3258.24	1000.00	30.69	69.31	**68.91**	**3.04**
	Bird 11	17.00	2.00	19.00	3394.00	3036.74	1110.00	36.55	63.45		
	Bird 12	21.00	1.00	22.00	3394.00	3239.73	843.50	26.04	73.96		

Supplementary Table S5. **Raw data for calculating nicotine degradation efficiency from sunbirds' excreta**, 24 and 72 h after nicotine addition to the food of birds with undisturbed microbiota (control, G2a) and birds with dysbiotic microbiota (G2b). Nicotine concentrations were measured by LC-MS analysis. n = 3 birds for each group treatment (see also Fig. 1 and Fig. 2). SEM, standard error of the mean; Conc., concentration.

**Nicotine, 24 h incubation**											
**Treatment**	**Bird ID**	**Food intake (ml)**	**Water intake (ml)**	**Total intake (ml)**	**Initial conc. of nicotine in food (ng/ml)**	**Total nicotine intake conc. (ng/ml)**	**Conc. of nicotine in excreta (ng/ml)**	**Nicotine left in excreta (%)**	**Nicotine degraded (%)**	**Average Nicotine degraded (%)**	**SEM**
**G2a** Control	Bird 1	15.00	1.50	16.50	332.00	301.82	174.50	57.82	42.18	**44.54**	**2.71**
	Bird 2	24.00	1.00	25.00	332.00	318.72	186.50	58.52	41.48		
	Bird 3	20.00	3.00	23.00	332.00	288.70	144.50	50.05	49.95		
**G2b** +Antibacterial agents	Bird 4	14.00	2.00	16.00	332.00	290.50	130.00	44.75	55.25	**31.77**	**11.76**
	Bird 5	17.00	2.00	19.00	332.00	297.05	233.50	78.61	21.39		
	Bird 6	20.00	4.00	24.00	332.00	276.67	225.00	81.33	18.67		
**Nicotine, 72 h incubation**											
**Treatment**	**Bird ID**	**Food intake (ml)**	**Water intake (ml)**	**Total intake (ml)**	**Initial conc. of nicotine in food (ng/ml)**	**Total nicotine intake conc. (ng/ml)**	**Conc. of nicotine in excreta (ng/ml)**	**Nicotine left in excreta (%)**	**Nicotine degraded (%)**	**Average Nicotine degraded (%)**	**SEM**
**G2a** Control	Bird 1	15.00	1.50	16.50	332.00	301.82	176.00	58.31	41.69	**52.79**	**7.24**
	Bird 2	24.00	1.00	25.00	332.00	318.72	158.50	49.73	50.27		
	Bird 3	20.00	3.00	23.00	332.00	288.70	97.00	33.60	66.40		
**G2b** +Antibacterial agents	Bird 4	14.00	2.00	16.00	332.00	290.50	119.50	41.14	58.86	**34.06**	**12.40**
	Bird 5	17.00	2.00	19.00	332.00	297.05	233.00	78.44	21.56		
	Bird 6	20.00	4.00	24.00	332.00	276.67	216.50	78.25	21.75		
